# Can liquid-based preparation substitute for conventional smear in thyroid fine-needle aspiration? A systematic review based on meta-analysis

**DOI:** 10.1530/EC-17-0165

**Published:** 2017-10-10

**Authors:** Yosep Chong, Soon-Jin Ji, Chang Suk Kang, Eun Jung Lee

**Affiliations:** 1 Department of Hospital PathologyCollege of Medicine, The Catholic University of Korea, Seoul, Republic of Korea; 2 Medical LibraryThe Catholic University of Korea, Seoul, Republic of Korea

**Keywords:** thyroid gland, fine-needle aspiration biopsy, liquid-based preparation, liquid-based cytology, meta-analysis

## Abstract

**Objective:**

Conventional smear (CS) using fine-needle aspiration cytology (FNAC) has been established as the test of choice for diagnosing thyroid lesions, despite low sample adequacy and inter-individual variations. Although a liquid-based preparation (LBP) technique has been recently applied to overcome these limitations, its clinical utility and its accuracy over CS are controversial. This study aimed to determine the true sensitivity and specificity of LBP in thyroid FNAC by meta-analysis.

**Design:**

Systematic review with meta-analysis.

**Methods:**

We searched major electronic databases (MEDLINE, EMBASE, Cochrane library, Google Scholar) with queries of ‘thyroid’, ‘LBP’ and ‘liquid-based cytology’. Original articles including cytohistologic correlation data comparing the accuracy of any LBP technique, such as ThinPrep, SurePath and Liqui-Prep, with CS were included for qualitative meta-analysis and preparation of synthesized reporter-operating curves (sROC).

**Results:**

A total of 372 studies were screened and 51 original articles were eligible for full-text review; finally, 24 studies were chosen for the meta-analysis. Average sample inadequacy was significantly lower in two mainstream LBP methods (ThinPrep and SurePath) than CS. Specificity and sensitivity by sROC were similar or slightly superior for LBP vs CS. Various cytomorphologic changes by each method have been reported.

**Conclusions:**

Although a learning curve is essential for adapting to the cytomorphologic features of the LBP technique, our results support the use of two mainstream LBPs alone in thyroid FNAC that LBP will increase the sample adequacy and reduce the workload with similar accuracy. More data and further evaluation are needed for the other LBP methods.

## Introduction

Conventional smear (CS) using fine-needle aspiration cytology (FNAC) has been well established during the last few decades as the diagnostic test of choice for making initial diagnosis and treatment plans for thyroid lesions (1). It has been widely accepted as a primary diagnostic tool owing to its simplicity, safety, possibility of repetition and cost-effectiveness. The major technical limitations in this test are those that occur during the smearing procedure: first, blood-obscuring background generated by abundant vasculature of thyroid lesions; second, poor cellularity due to extensive fibrosis or the cystic nature of the lesion itself; and third, person-to-person variation in the smearing technique often leading to dry artifacts. These problems result in a number of inadequate samples for making a proper diagnosis and cause a decrease in overall efficacy. In fact, many studies have reported a quite high rate of inadequate samples in thyroid FNAC using CS, up to 50.5% (2). Therefore, such limitations derived from the nature of the thyroid lesions and the essential parts of the procedure that involve various medical personnel have been a major hindrance to overcome.

Liquid-based preparation (LBP), or thin-layer preparation, was first introduced for gynecologic cervical smears that have similar limitations. Two major systems approved by the United States Food and Drug Administration, ThinPrep (Hologic, Marlborough, MA, USA) and SurePath (BD Diagnostics-TriPath Imaging, Burlington, NC, USA), are basically designed to reduce such variations and artifacts and are intended to produce representative, standardized smears by an automated process. Both techniques consist of collection of the aspirates in specially developed liquid fixative; followed by removal of cell debris, red blood cells and inflammatory cells; homogenization by vortexing and finally a sampling and slideproducing step either by vacuum application or a sedimentation method.

Over 25 years of wide spread use, the diagnostic utility of LBP in gynecologic samples has been relatively well-verified, clarifying its strong and weak points. It provides standardized slides of homogenous cellular smears with well-preserved cell morphology resulting in clearer visualization, shorter interpretation time and more reproducible results among various cytotechnicians and pathologists. In particular, dispersing cell clusters into single cells during the homogenizing step in LBP is an important strong point for gynecologic samples in which cell overlapping is a major hindrance for accurate interpretation.

In terms of application to thyroid FNAC samples, however, where the shapes of cell clusters and the nature of the background are valuable for accurate diagnosis, conflicting results on the diagnostic utility of LBP have been reported. These might be attributable to the diversity of the subject population, subtle differences in detailed procedures and the infancy of application of this technology in this specialized field.

However, many studies have been designed and conducted under pressure to some extent to favor a certain LBP product, potentially leading to biased results, and this should not be neglected. For example, many studies applied different interpretation criteria for sensitivity and specificity that were favorable for their preferred conclusion. Although many investigators now agree that application of LBP to thyroid FNACs is acceptable, to what extent we can trust the results of LBP, whether it is okay to use LBP alone or whether LBP should be applied in combination with CS are aspects that are not clear.

In this study, we performed a systematic review and meta-analysis of the comparative studies of LBP and CS, mainly on ThinPrep and SurePath methods, conducted in thyroid FNACs to draw less biased results and a statistically convincing conclusion on the diagnostic accuracy and utility of LBP in thyroid FNACs.

## Subjects and methods

### Subjects

This study was approved by the Institutional Review Board of The Catholic University of Korea, College of Medicine (SC15OISbib4). We searched major electronic databases from January 1, 1990 to January 5, 2015 for relevant articles published in medical journals with abstracts written in English. Included databases were MEDLINE (PubMed), Cochrane Library, EMBASE and Google Scholar. There were 7 LBP methods included in the search: ThinPrep, SurePath (also known as AutoCyte PREP), Liqui-PREP (LGM-International, FL, USA), CellPrepPlus (Biodyne, Seongnam, Korea), Cell & Tech (Cell & Tech Bio, Seoul, Korea), EasyPrep (YD Diagnostics Corp., Seoul, Korea) and HuroPath (formerly known as E-Prep, CelltraZone, Seoul, Korea). The queries used were: ‘(‘Thyroid gland’ (MeSH Term) OR ‘Thyroid gland’ (Text Word)) AND (‘liquid-based preparation’ (Text Word) OR ‘liquid-based cytology’ (Text Word) OR ‘ThinPrep’ (Text Word) OR ‘SurePath’ (Text Word) OR ‘AutoCyte PREP’ (Text Word) OR ‘Liqui-Prep’ (Text Word) OR ‘CellPrepPlus’ (Text Word) OR ‘Cell & Tech’ (Text Word) OR ‘EasyPrep’ (Text Word) OR ‘E-Prep’ (Text Word))’ for PubMed, ‘thyroid gland’/exp AND (‘liquid-based preparation’/exp OR ‘liquid based cytology’ OR ‘thinprep’/exp OR ‘surepath’/exp OR ‘autocyte prep’/exp OR ‘liqui-prep’/exp OR ‘cellprepplus’/exp OR ‘cell & tech’/exp OR ‘easyprep’/exp OR ‘e-prep’/exp) for Embase, ‘allintitle: thyroid AND (‘thinprep’ OR ‘surepath’ OR ‘autocyte prep’ OR ‘liqui-prep’ OR ‘cellpreppro’ OR ‘cell & tech’ OR ‘easyprep’ OR ‘e-prep’ OR ‘liquid based preparation’ OR ‘liquid based cytology’’ for Google Scholar. All similar possible word variations were also searched. The attained records were retrieved and managed with EndNote X7.2.1 (Bld 10136, Thomson Reuters, New York, NY, USA).

### Study selection, reviewing and data retrieving

**Figure 1:**
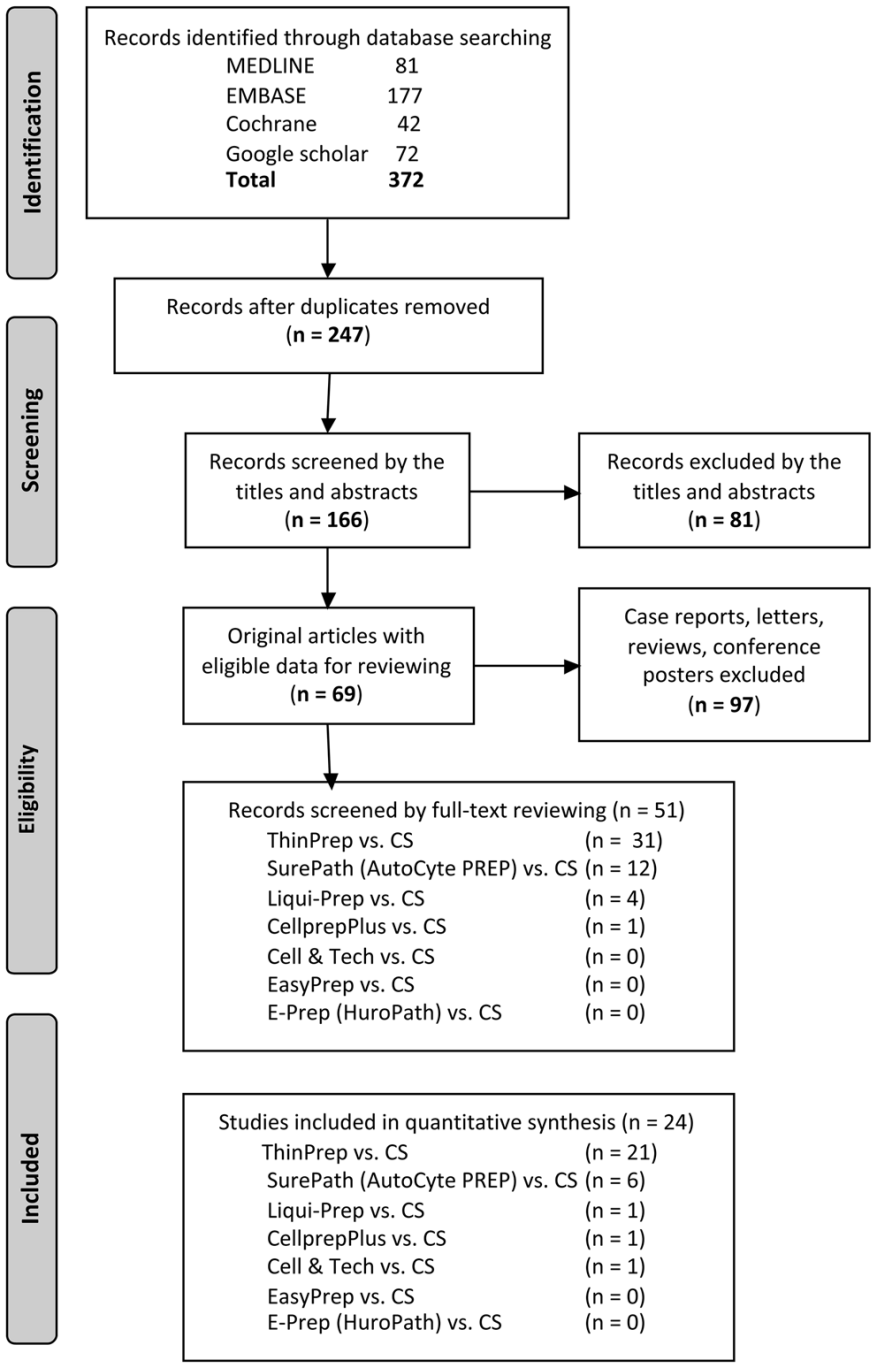
Schematic illustration of the selection steps for reviewing candidate studies.

The process of study selection and reviewing is depicted in Fig. 1. After the initial search, any duplicates were removed from the results. Then, the title and abstracts of the records were screened by two independent reviewers (Y Chong and E J Lee). Case reports, letters, reviews, conference proceedings and posters were excluded. Any original studies with cytohistological correlation data were included for full-text reviewing and only a subset of the studies with eligible data was used for quantitative analysis. In addition, the cited references in each study were manually searched and reviewed to identify any additional relevant studies.

To apply the same standardized criteria for determining sensitivity and specificity, data from each study were retrieved and properly treated for quantitative analysis. Based on the treatment guidelines for thyroid lesions after FNAC, follicular neoplasms (FN) or Hurthle cell neoplasms (HCN) were considered to require surgical resection. Thus, we applied a tentative definition of positive, false positive, negative, and false negative solely based on the need for surgical resection. For example, FNAC results of FN or HCN with a histologic diagnosis of nodular hyperplasia (NH) or other benign lesions were tentatively considered as false-positive results, while the cases with histologic diagnosis of papillary carcinoma (PTC) were tentatively considered as positive results although the cytomorphologic features of FN/HCN and PTC are different. Likewise, FNAC results of atypia of undetermined significance (AUS) or benign follicular nodule with histologic diagnosis of FN or HCN were tentatively considered as false negative while the cases with NH or thyroiditis were tentatively considered as negative results. We hypothesized that the proportion of tentatively categorized false-positive or false-negative cases may be similar regardless of CS or LBP. Detailed interpretation criteria are shown in Table 1. Similar possible terminological variations among studies were recategorized into the relevant subcategories.

**Table 1:** Detailed tentative criteria for interpretation of FNAC results compared with histologic diagnosis.

**Interpretation**	**FNAC results of CS or LBP**	**Histologic diagnosis**
Positive	Malignancy	PTC
	Suspicious for malignancy	Other types of primary thyroidal malignancy including lymphoma
	FN (suspicious for)	FA*/FC
	HCN (suspicious for)	HCA*/HCC
False positive	Malignancy	Nodular hyperplasia/colloid cyst
	Suspicious for malignancy	
	FN (suspicious for)	Nodular hyperplasia/colloid cyst
	HCN (suspicious for)	
Negative	Benign follicular nodule	Nodular hyperplasia/colloid cyst
	AUS/FLUS*	Thyroiditis (Hashimoto’s/lymphocytic/granulomatous)
False negative	Benign follicular nodule	PTC
		Other types of primary thyroidal malignancy including lymphoma
	Benign follicular nodule	FA*/FC
		HCA*/HCC

AUS, atypia of undetermined significance; FA, follicular adenoma; FC, follicular carcinoma; FLUS, follicular lesion of undetermined significance; HCA, Hurthle cell adenoma; HCC, Hurthle cell carcinoma; PTC, papillary thyroid carcinoma.

### Quality assessment of diagnostic accuracy studies

To assess the quality of the studies included in the meta-analysis, we incorporated the revised quality assessment of diagnostic accuracy studies, QUADAS-2, developed by Whiting *et al*. (3). QUADAS-2 consists of four key domains: patient selection, index test, reference standard and flow and timing. A few signaling questions relevant to the risk of bias and the applicability in the index test domain were designed and added to the quality assessment as follows: Was a standardized classification system used? Was any risk of bias according to individual variation in the obtaining technique avoided? Was any risk of bias during data transformation and utilization avoided as much as possible?

Two independent reviewers (Y Chong and EJ Lee) reviewed the included studies using the modified QUADAS-2 and made a judgment on the risk of bias and the applicability of each domain. The results were tabulated and summarized after a discussion about the studies with discrepant assessments. The final meta-analysis was performed after the exclusion of any studies with a high risk of bias or concerns about bias in any of the domains.

### Data extraction and analysis

The weighted average of sample inadequacy was calculated for each LBP method and CS. To determine the statistical significance, the weighted average difference was calculated in the studies that evaluated both LBP and CS using the same sample. A *P* value of less than 0.05 was defined as statistically significant. The reported sample inadequacy was categorized by the year of publication and compared by the mode of sampling method.

## Results

### Study selection, reviewing and data retrieving

The inclusion/exclusion process during the screening and selection steps is summarized in Fig. 1. A total of 372 papers were identified by the database search (81 in MEDLINE, 177 in EMBASE, 42 in Cochrane library and 72 in Google Scholar). After excluding 125 duplicates, a total of 247 records were screened by titles and abstracts. After 81 records were removed, 166 studies were subjected to further evaluation. Ninety-seven records of case reports, letters, reviews and conference proceedings were excluded, and only 51 studies were eligible for full-text reviewing (31 on TP, 12 on SP, 4 on Liqui-Prep, 1 on CellprepPlus, 1 on Cell & Tech, none on EasyPrep or E-prep). Among these, only 24 studies were eligible for data retrieval and qualitative synthesis (21 on TP, 6 on SP, 1 on Liqui-Prep, 1 on CellprepPlus, 1 on Cell & Tech, none on EasyPrep or E-prep).

### Sample inadequacy of LBP

The average sample inadequacy using all of the case data from the studies using either LBP alone, or LBP and CS or LBP and a combined method, are summarized in Table 2 (4, 5, 6, 7, 8, 9, 10, 11, 12, 13, 14, 15, 16, 17, 18, 19, 20). The average sample inadequacy in TP studies (17 studies, 24,880 cases) was 16.4% in the cases processed by TP, 33.4% by CS and 7.7% by a combined method (Table 2A). Average sample inadequacy in the SP studies (4 studies, 1706 cases) was 18.9% in the cases processed by SP, 12.6% by CS and 4.0% by a combined method (Table 2B) (21, 22, 23, 24). Only one study each was included for calculating average sample inadequacy for Liqui-PREP (25), CellprepPlus (26) and the Cell and Tech method (27) (Table 2C, D and E).

**Table 2:** Reported sample adequacy (weighted average) of the studies using a LBP and a CS/combined method.

**Year**	**Authors**	**Total cases**	**Inadequacy** (case number/%)						Sampling method
			TP		CS		TP + CS		
(A) ThinPrep vs conventional smear									
2000	Scurry *et al*. (4)	728	401	40.9	327	50.5	–	–	Direct to vial
2001	Afify *et al*. (5)	209	82	11.0	127	20.5	–	–	Syringe rinsing
2001	Nasuti *et al*. (6)	66	33	27.3	–	–	33	15.2	Consultation slide
2003	Cochand-Priollet *et al*. (7)	240	120	22.5	120	8.3	–	–	Splitting
2004	Tulecke *et al*. (8)	115	115	6.1	–	–	–	–	Direct to vial
2006	Fadda *et al*. (9)	2006	2006	11.3	–	–	–	–	Direct to vial
2006	Malle *et al*. (10)	744	459	3.9	285	8.9	–	–	Syringe rinsing
2007	Michael *et al*. (11)	218	218	33.0	–	–	–	–	Direct to vial
2008	Cavaliere *et al*. (12)	7750	3875	32.3	3875	36.5	–	–	Double sampling
2008	Saleh *et al*. (13)	290	145	24.1	145	37.2	–	–	Syringe rinsing
2008	Stamataki *et al*. (14)	252	252	4.0	–	–	–	–	Direct to vial
2010	Ardito *et al*. (15)	353	–	–	–	–	353	1.1	Syringe rinsing
2011	Gheri *et al*. (16)	2518	2518	9.4	–	–	–	–	Direct to vial
2011	Luu *et al*. (17)	4101	2101	8.2	–	–	2000	8.7	Syringe rinsing
2012	Chang *et al*. (18)	4290	2523	12.6	1767	25.9	–	–	Direct to vial
2013	Mastorakis *et al*. (19)	1000	1000	4.1	–	–	–	–	Direct to vial
Total		24,480	15,848	16.4	6646	33.4	2386	7.7	

**Year**	**Authors**	**Total cases**	**Inadequacy** (case number/%)						**Sampling method**
			SP		CS		SP + CS		
(B) SurePath vs conventional smear									
2007	Kim *et al*. (21)	344	172	9.3	172	20.9	–	–	Direct to vial
2008	Jung *et al*. (22)	386	193	5.2	193	6.2	–	–	Syringe rinsing
2009	Sidiropoulos *et al*. (23)	264	–	–	121	11.0	143	4.0	Syringe rinsing
2011	Geers *et al*. (24)	712	712	25.0	–	–	–	–	Direct to vial
Total		1706	1077	18.9	486	12.6	143	4.0	

**Year**	**Authors**	**Total cases**	**Inadequacy** (case number/%)						**Sampling method**
			LP		CS		LP + CS		
(C) Liqui-PREP vs conventional smear									
2012	Tetikkurt *et al*. (25)	378	189	4.2	189	4.2			Double sampling
Total		378	189	4.2	189	4.2			

**Year**	**Authors**	**Total cases**	**Inadequacy** (case number/%)						**Sampling method**
			CP		CS		CP + CS		
(D) CellprepPlus vs conventional smear									
2011	Koo *et al*. (26)	396	198	48.5	198	16.2			Double sampling
Total		396	198	48.5	198	16.2			

**Year**	**Authors**	**Total cases**	**Inadequacy** (case number/%)						**Sampling method**
			CT		CS		CT + CS		
(E) Cell and Tech vs conventional smear									
2013	Lee *et al*. (27)	153	70	22.9	83	1.2			splitting
Total		153	70	22.9	83	1.2			

CP, CellprepPlus; CS, conventional smear; LBP, liquid-based preparation; LP, Liqui-PREP; TP, ThinPrep; SP, SurePath.

Sample inadequacy using the case data of the comparative studies of LBP and CS is summarized in Table 3 (4, 5, 7, 10, 12, 13, 18). Average sample inadequacy in TP studies (7 studies, 14,251 cases) was significantly lower in TP (24.0%) than CS (33.4%) (*P* < 0.01). Likewise, average sample inadequacy in SP studies (2 studies, 730 cases) was significantly lower in SP (7.1%) than CS (13.2%) (*P* < 0.02), although the number of included studies was limited.

**Table 3:** Difference in sample inadequacy between LBP and CS (weighted average difference).

**Year**	**Authors**	**Total cases**	**Inadequacy** (case number/%)				**Sampling method**
			TP		CS		
(A) ThinPrep vs CS							
2000	Scurry *et al*. (4)	728	401	40.9	327	50.5	Direct to vial
2001	Afify *et al*. (5)	209	82	11.0	127	20.5	Syringe rinsing
2003	Cochand-Priollet *et al*. (7)	240	120	22.5	120	8.3	Splitting
2006	Malle *et al*. (10)	744	459	3.9	285	8.9	Syringe rinsing
2008	Cavaliere *et al*. (12)	7750	3875	32.3	3875	36.5	Double sampling
2008	Saleh *et al*. (13)	290	145	24.1	145	37.2	Syringe rinsing
2012	Chang *et al*. (18)	4290	2523	12.6	1767	25.9	Direct to vial
Total	14,251	7605	24.0	6646	33.4	*P* < 0.01	

**Year**	**Authors**	**Total cases**	**Inadequacy** (case number/%)				**Sampling method**
			SP		CS		
(B) SurePath vs CS							
2007	Kim *et al*. (21)	344	172	9.3	172	20.9	Direct to vial
2008	Jung *et al*. (22)	386	193	5.2	193	6.2	Syringe rinsing
Total	730	365	7.1	365	13.2	*P* < 0.02	

(A) TP vs CS, (B) SP vs CS.

CS, conventional smear; LBP, liquid-based preparation; SP, SurePath; TP, ThinPrep.

**Figure 2:**
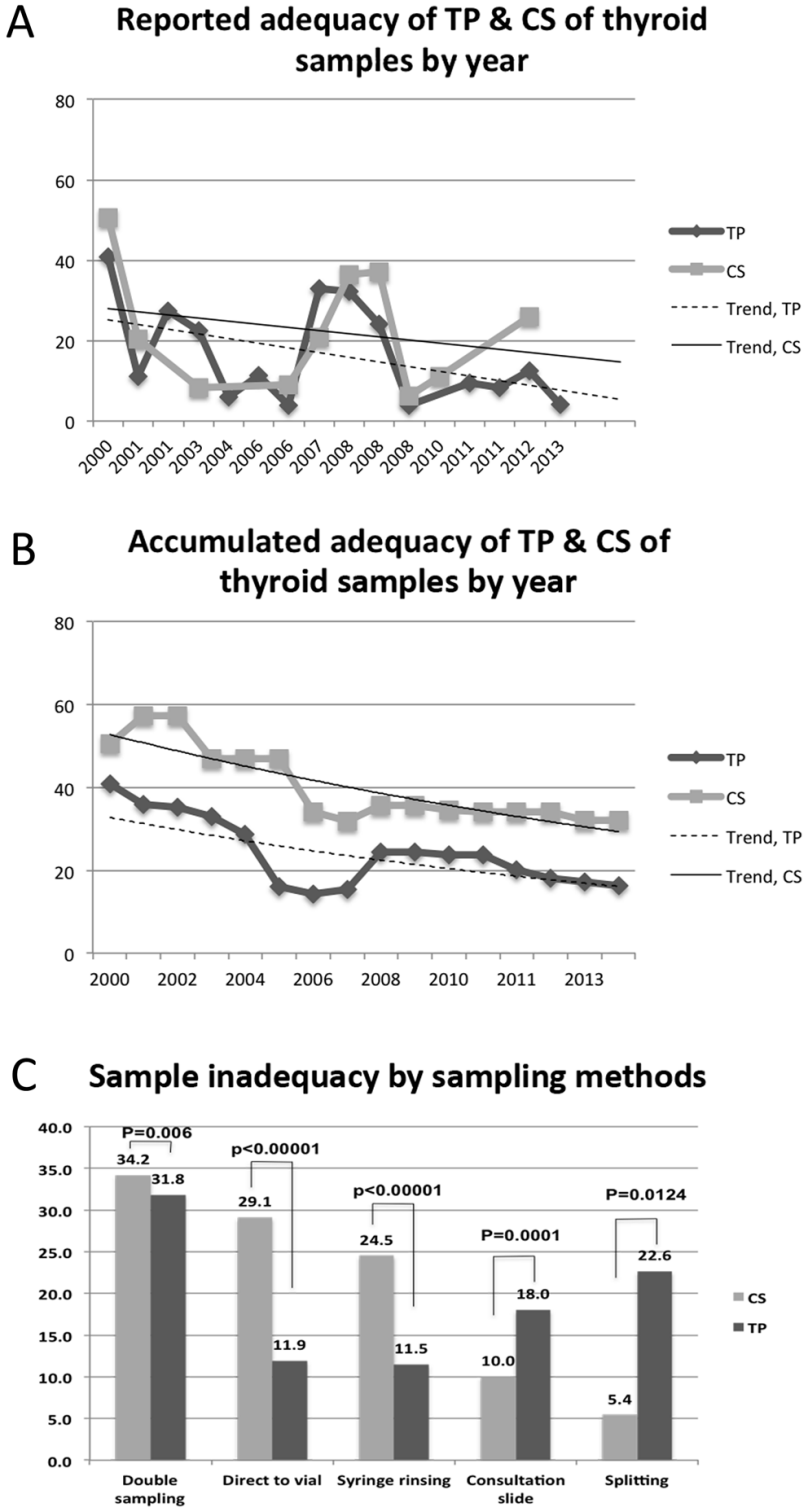
Sample inadequacy rate of TP and CS. Trends of reported inadequacy (A) and accumulated inadequacy (B) of TP and CS of thyroid samples by year, sample inadequacy rate by various sampling methods (C). CS, conventional smear; TP, ThinPrep.

Sample inadequacy of TP studies gradually decreased from the first dates of publication to the more recent ones and the inadequacy trend line of TP was slightly lower than that of CS (Fig. 2A). The accumulated inadequacy of TP studies more clearly demonstrates this finding (Fig. 2B). Sample inadequacy was significantly lower by TP than CS in samples collected by double sampling or syringe rinsing or directly collected to vial from the different patients (during the different periods) (Fig. 2C). However, sample inadequacy was significantly higher in TP than CS in the consultation slide or the samples collected by splitting (Fig. 2C).

### Sensitivity and specificity of LBP

**Figure 3:**
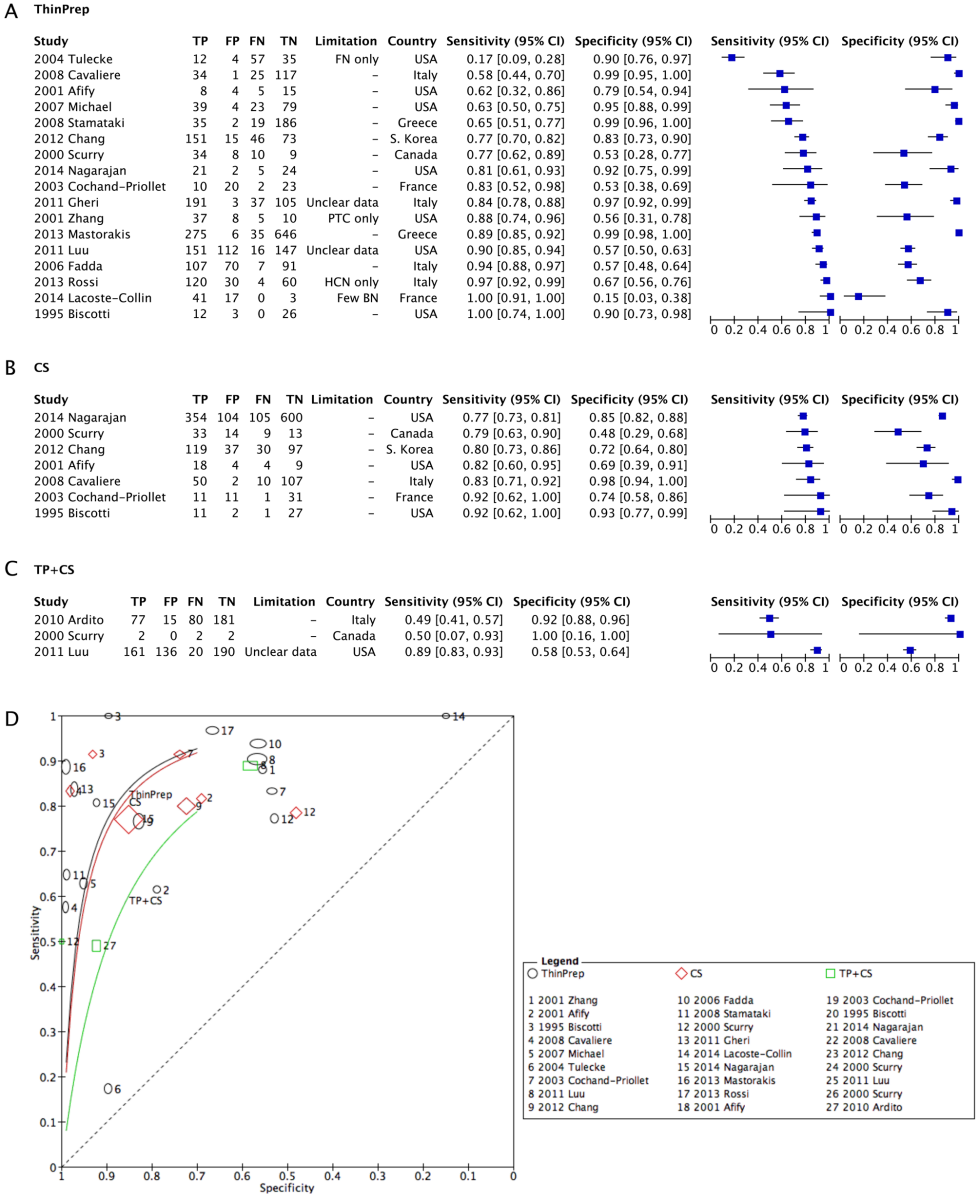
Coupled forest plots of the studies using TP (A), CS (B) and combined TP and CS (C), and a corresponding sROC (D). CS, conventional smear; sROC, synthesized reporter operating curve; TP, ThinPrep.

A coupled forest plot of sensitivity and specificity of 17 TP studies is shown in Fig. 3A (4, 5, 7, 8, 9, 11, 12, 14, 15, 16, 17, 18, 19, 20, 28, 29, 30, 31). There was no obvious relationship between sensitivity and specificity among 17 studies using TP. Sensitivity and specificity of CS showed more homogeneous results than those of TP because TP data in some studies were limited by sample type and data quality (Fig. 3A). A forest plot of the combined TP and CS studies showed heterogeneity because of the limited number of included studies and the limited data quality of the included studies (Fig. 3C) (*Q* value). The sROC using these data showed similar curves between TP and CS; more precisely, a slightly higher curve for TP, showing a significant difference in specificity and sensitivity between TP and CS in thyroid FNAC (Fig. 3D). Although the sROC of the combined TP and CS was lower than the others, it should be interpreted with caution because it was based on data derived from a limited number of studies.

**Figure 4:**
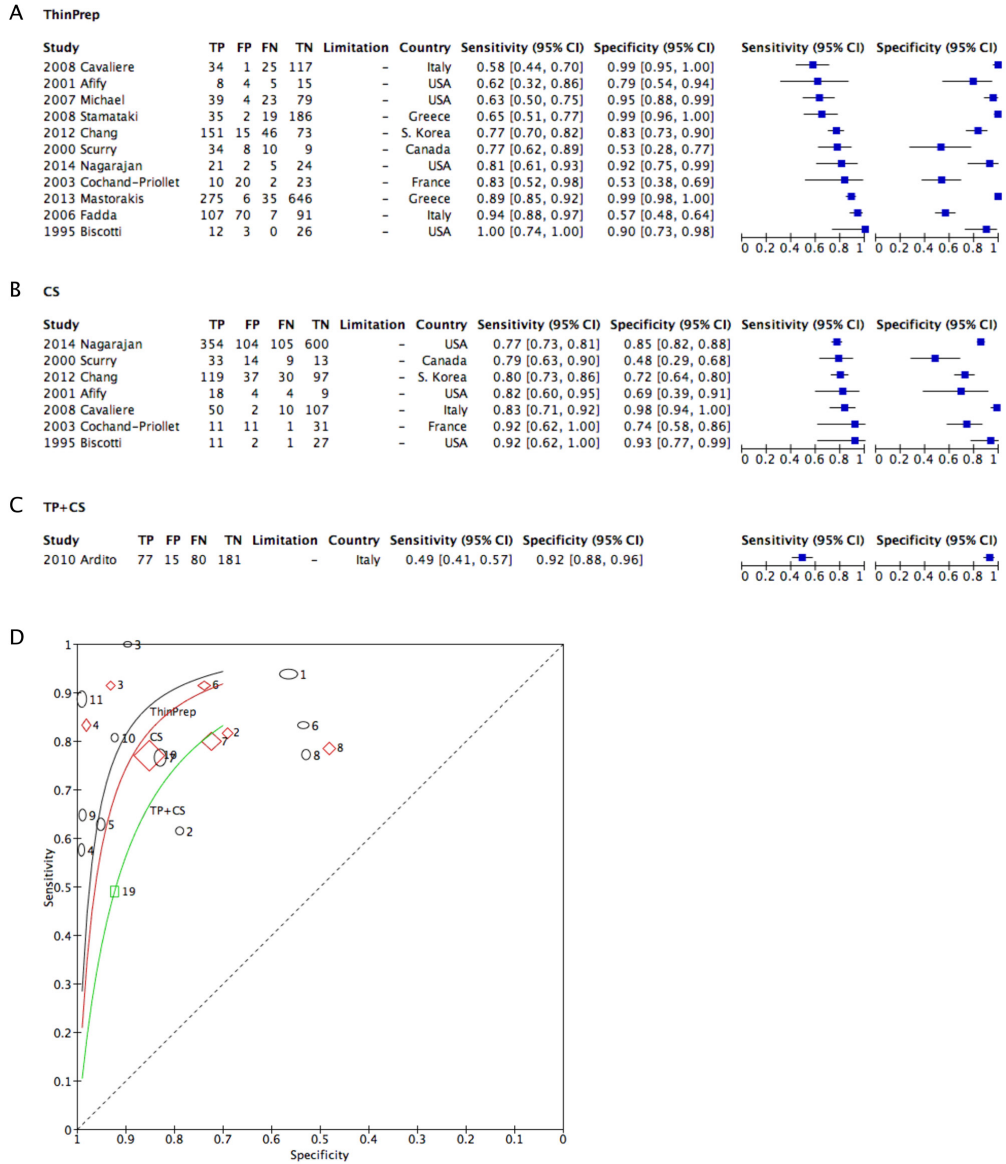
Coupled forest plots after exclusion of limited studies using TP (A), CS (B) and combined TP and CS (C), and a corresponding sROC (D). CS, conventional smear; sROC, synthesized reporter operating curve; TP, ThinPrep.

A forest plot of TP after exclusion of studies of limited quality showed more homogenous results (Fig. 4A) (4, 5, 7, 9, 11, 12, 14, 15, 18, 19, 20, 31). The heterogeneity of studies of TP and CS was similar (based on the *Q* value). Only one study was included in this analysis for combined TP and CS (Fig. 4C). The sROC derived from these studies showed similar but slightly higher curves for TP than the previous sROC (Fig. 4D).

**Figure 5:**
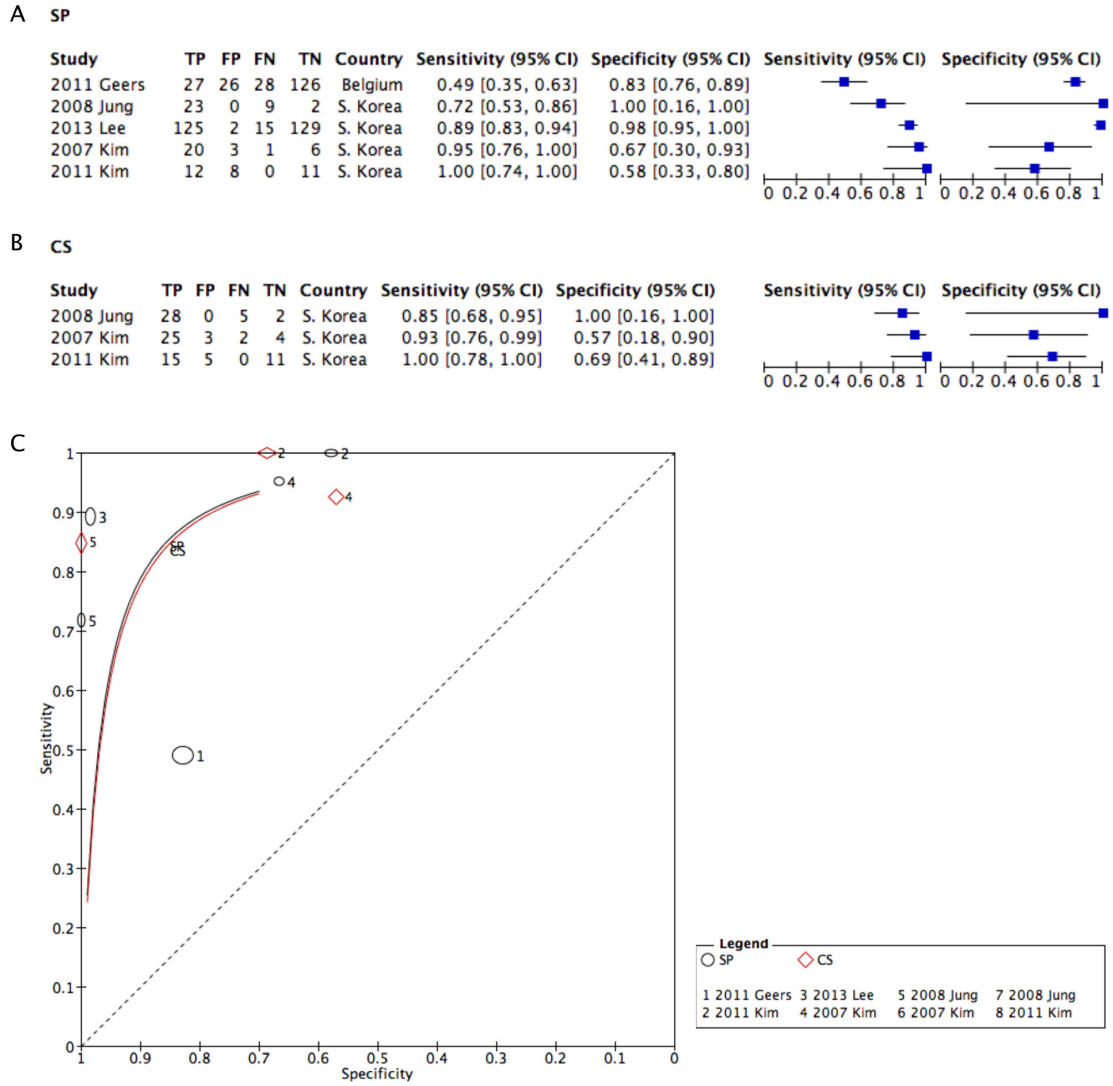
Coupled forest plots after exclusion of limited studies using SP (A), CS (B) and a corresponding sROC (C). CS, conventional smear; SP, SurePath; sROC, synthesized reporter operating curve.

For SP, a forest plot drawn from SP and CS is depicted in Fig. 5A and B, and it showed moderate heterogeneity (*Q* value) (21, 22, 24, 32, 33). A sROC using 5 SP studies and 3 CS studies showed curves with similar sensitivity and specificity (Fig. 5C).

### Quality assessment of diagnostic accuracy studies

Quality assessments of the included studies are summarized in Table 4 and Supplementary Fig. 1 (see section on supplementary data given at the end of this article). Most studies in the final meta-analysis had a low risk of bias or concern in each domain according to risk of bias or applicability, which represents a relatively high level of credibility in the results of the meta-analysis. Three studies showed an uncertain risk of bias or concern in one domain among the TP studies. Only one study among SP studies showed uncertain concern in the applicability during the patient selection.

**Table 4:** Results of QUADAS-2 of the studies included in the final meta-analysis.

	**Risk of bias**				**Applicability concerns**		
	Patient selection	Index test	Reference standard	Flowing and timing	Patient selection	Index test	Reference standard
TP studies							
1995 Biscotti *et al*. (31)	L	L	L	L	L	L	L
2001 Afify *et al*. (5)	L	?	L	L	L	L	L
2003 Cochand-Priollet *et al*. (7)	L	L	L	L	L	L	L
2006 Fadda *et al*. (9)	L	L	L	L	L	L	L
2007 Michael *et al*. (11)	L	L	L	L	L	L	L
2008 Cavaliere *et al*. (12)	L	L	L	L	L	L	L
2008 Stamataki *et al*. (14)	L	L	L	L	L	L	L
2010 Ardito *et al*. (15)	L	L	L	L	L	L	L
2012 Chang *et al*. (18)	L	L	L	L	L	L	L
2014 Mastorakis *et al*. (19)	L	L	?	L	L	L	L
SP studies							
2007 Kim *et al*. (21)	L	L	L	L	L	L	L
2008 Jung *et al*. (22)	L	L	L	L	L	L	L
2011 Geers *et al*. (24)	L	L	L	L	L	L	L
2011 Kim *et al*. (33)	L	L	L	L	?	L	L
2013 Lee *et al*. (32)	L	L	L	L	L	L	L

L, low risk; H, high risk; ?, unclear risk; QUADAS-2, revised quality assessment of diagnostic accuracy studies; SP, SurePath; TP, ThinPrep.

### Morphologic characteristics of LBP

Morphologic parameters could not be compared using meta-analysis because each study applied a customized strategy that could not be easily standardized for meta-analysis. Major morphologic characteristics of each LBP compared to CS described by a few key studies are summarized in Table 5 (8, 18, 27, 28, 29, 34, 35).

**Table 5:** Major morphologic characteristics of liquid-based preparation compared to conventional smear reported in the literature.

**Year**	**Authors**	**LBP method**	**Morphologic characteristics**
2001	Zhang *et al*. (28)	TP	Intranuclear inclusion, papillary and/or sheet arrangements, nuclear grooves, powdery chromatin and nuclear molding are the most powerful features in LBP for differential diagnosis of PTC from others
2003	Cochand-Priollet *et al*. (7)	TP	The colloids often occur in small dense droplets rather than in a film. Nuclei tend to appear smaller but the nuclear details, especially the nuclear membrane, the chromatin and the nucleoli are more easily observed. In cases of PTC on TP slides, nuclear cytoplasmic inclusions are rare but nuclear grooves and ground-glass nuclei are frequently observed. Oncocytic cells have a pale cytoplasm mostly lacking blue granules on TP. The diagnosis of Hashimoto’s thyroiditis is more difficult on TP slides than on CS because of the limited number of lymphocytes
2004	Tulecke *et al*. (8)	TP	Large colloid fragments, tissue paper-like material, cystic change and monolayered sheets of follicular cells in LBP are associated with macrofollicular or mixed architecture on histology
2010	Chae *et al*. (34)	SP	LBP showed higher cellularity, better 3D configuration, more follicle patterns and dispersed single cells
2012	Chang *et al*. (18)	TP	LBP showed better preserved nuclear details, a cleaner background and fewer large papillae than CS
2012	Tetikkurt *et al*. (25)	Liqui-PREP	The most important and specific features for papillary carcinoma are the presence of intranuclear inclusions and grooves. Nuclear irregularity is the crucial hallmark for the differentiation of follicular neoplasm and malignant lesions. The presence of nucleoli and regular nucleus may appear as other outstanding features for the diagnosis of follicular neoplasm
2013	Chung *et al*. (35)	SP	Hobnail features in papillary carcinoma are often associated with cytoplasmic vacuole, background macrophages and lymph node metastasis
2013	Rossi *et al*. (29)	TP	Large oncocytic cells with a discohesive pattern are more common in malignant oncocytic/Hurthle cell neoplasms
2013	Lee *et al*. (27)	Cell & Tech	Nuclear grooves and intranuclear inclusions are difficult to visualize on LBP
2016	Chong *et al*. (36)	EasyPrep	Compared to SP, EasyPrep allows easier fragmentation of the cell clusters, producing more 2-dimensional sheet-like clusters in benign cases, and clearer visibility for nuclear features of PTCs. The nuclear size is smaller on SP, which makes nuclear irregularity and grooves more evident. However, intranuclear inclusions are less obvious. Incompletely lysed red blood cells are more prominent in EasyPrep

CS, conventional smear; LBP, liquid-based preparation; PTC, papillary thyroid carcinoma; SP, SurePath; TP, ThinPrep.

## Discussion

This study demonstrated that the clinical utility of LBP is almost the same or marginally better than that of CS for thyroid FNAC in terms of sample adequacy, sensitivity and specificity.

### Sample inadequacy of LBP

The sample adequacy was significantly superior for two mainstream LBP methods (ThinPrep and SurePath) than CS for most sampling methods (Table 3). More data on sample adequacy are needed for the newly developed techniques such as Liqui-PREP, CellprepPlus and the Cell and Tech method (Table 2). We can see clearly that the sample adequacy is getting better over time after the introduction of LBP for thyroid FNAC (Fig. 2A and B). This must be due to a learning curve of the new technology. This trend suggests that the learning curve has reached a stage of maturity for the new technology.

As expected, sample adequacy was better for LBP (TP) than CS using sampling methods where relatively equal amounts of sample content might be distributed to LBP and CS, such as double sampling, direct to vial (different samples), and syringe rinsing methods. Sample adequacy using consultation slides and sample splitting methods showed better adequacy for CS than TP. This can be explained by the fact that most consultation slides contain samples from the primary screening laboratory that might contain generally lower cellularity. Sample splitting is a limited method inevitably producing disproportionate samples used for LBP and CS, as many prior studies have shown. Combined LBP and CS methods showed much lower rates of sample inadequacy than LBP or CS alone for both TP and SP studies, although the difference was not statistically significant (Table 2).

### Sensitivity and specificity of LBP

The sensitivity and specificity of LBP over CS using sROC showed similar or slightly better results for TP than CS (Fig. 3). The results were clearer after the exclusion of studies with poor or limited data quality (Fig. 4). The results were similar for SP studies as well (Fig. 5). For SP studies, there is a generalizability limitation because the included studies are all from either Belgium or South Korea.

On a side note to the results of meta-analysis, there were two studies that deal with cytohistological correlation data of other LBPs, one for CellprepPlus and one for EasyPrep (26, 36). The CellprepPlus study compared only 20 cases of CellprepPlus and CS, but it showed 100% sensitivity and specificity for CS and 71.4% sensitivity and 86.7% negative predictive value for CellprepPlus for the histologic correlation (data not shown) (26). The EasyPrep study compared only 28 and 26 cases of SP and EasyPrep and histologic diagnosis and showed 100% sensitivity and specificity for SP and 95.5% sensitivity and 80% negative predictive value for EasyPrep (36).

### Morphologic characteristics of LBP

The morphologic parameters were not feasible for meta-analytic comparison because it is generally thought that the standards of morphologic parameters vary widely among various sampling methods, LBP methods, study designs and investigators. However, we can summarize the general morphologic changes of LBP in thyroid FNAC compared to CS based on the consistent findings of most studies. In LBP, the nuclear size is smaller, the nuclear-to-cytoplasmic ratio is bigger, nucleoli are more prominent and nuclear membrane irregularity and nuclear grooves become more obvious. The cytoplasm is scantier in LBP. These changes are probably due to a lack of the smearing effect that can be a potential cause of dry or degenerative artifacts in CS. However, intranuclear pseudoinclusions are less evident on LBP than CS owing to the ingredient changes in the fixative solutions of LBP. Increases of 3-dimensional clusters and decreases of large papillae by fragmentation are other important features of LBP compared to CS. These can be understood to be a result of the homogenizing step of LBP. Therefore, LBP gives better cytomorphologic visibility for follicular clusters of the benign lesions while it loses the important papillary structure of PTCs. For follicular lesions, macrofollicular architecture, Hurthle cell changes and the presence of colloid (tissue-paper-like material) and macrophages serve as important features to suspect benign follicular lesions, which is similar in CS (8).

### Quality assessment of diagnostic accuracy studies

QUADAS-2 assessment of the included studies revealed that only a few studies had an uncertain risk of bias or concern in one domain, which means that the results of this meta-analysis are trustworthy. Furthermore, the results of the sROC curve before and after the exclusion of the studies with limited quality were consistent. From the beginning, we hypothesized that the simplified, tentative categorization of the cytopathological diagnosis into four groups according to the consequent surgical treatment plan (Table 1), true and false positive, or true and false negative, might not influence the results of the meta-analysis mathematically. However, there should be caution when applying the results of this study under some circumstances.

## Conclusion

Based on the results of this study, we conclude that it is reasonable for LBP to be substituted for CS of FNAC of thyroid lesions for the following reasons. First, the sample adequacy is statistically superior for LBP than CS. Second, the sensitivity and specificity of LBP was similar or slightly superior to that of CS. Third, although an educational period is essential and there are pros and cons of cytomorphologic features using LBP in thyroid FNAC, it does not seem to greatly affect the accuracy of the diagnosis itself. Therefore, it is okay to trust the results of any of the two major LBPs (TP and SP) for thyroid FNAC, even when it is performed alone without additional CS. However, additional data and further evaluation are needed for the other LBPs to confirm their results.

## Supplementary Material

Supporting Figure 1

## Declaration of interest

The authors declare that there is no conflict of interest that could be perceived as prejudicing the impartiality of the research reported.

## Funding

This work was supported by the research grant from Institute of Clinical Medicine Research in the Catholic University of Korea, Yeouido St. Mary’s Hospital.

## Author contribution statement

Y C designed the study, participated in screening, selection, and reviewing the references, data analysis and wrote the draft. S J J designed the query and search the databases. C S K reviewed the data analysis and revised the manuscript critically. E J L designed the study and participated in screening, selection, and reviewing the reference, data analysis and reviewed the final manuscript.
