# Supplementary figures and images for: Can liquid-based preparation substitute for conventional smear in thyroid fine-needle aspiration? A systematic review based on meta-analysis
Source: Endocr Connect. 2017 Oct 10;6(8):817–29. doi: 10.1530/EC-17-0165 (PMC5682413; doi:10.1530/EC-17-0165)

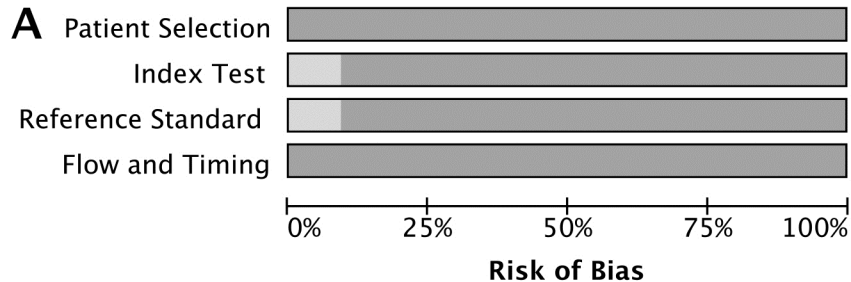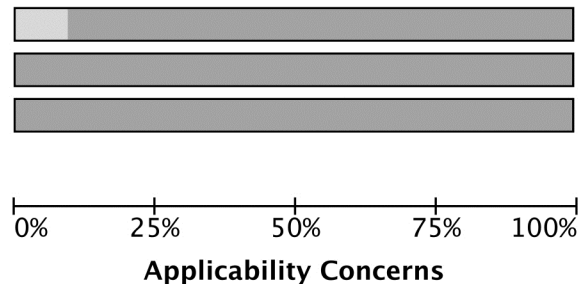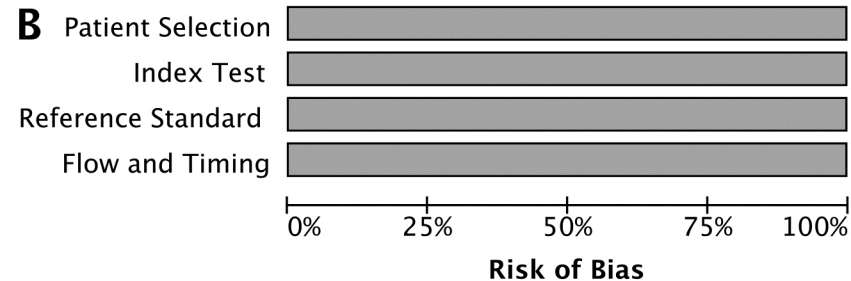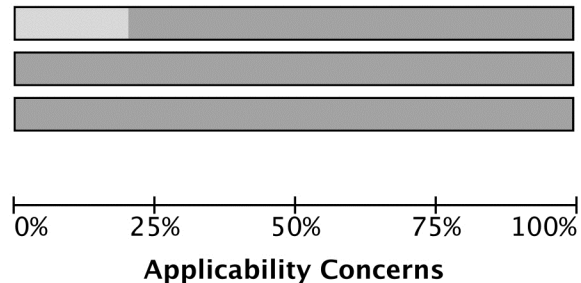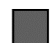

High

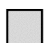

Unclear

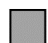

Low

Supplement: Supporting Figure 1 [file ec-6-817-s001.pdf]
